# Ability to form Sox17-induced hematopoietic cell clusters varies among distinct hematopoietic sites during development

**DOI:** 10.1186/s41232-026-00420-w

**Published:** 2026-04-23

**Authors:** Ayumi Itabashi, Yuki Yokoi, Kiyoka Saito, Ryota Tsukahara, Gerel Melig, Koya Azuma, Naoki Iizuka, Tetsuya Taga, Ikuo Nobuhisa

**Affiliations:** 1 Department of Stem Cell Regulation, Medical Research Laboratory, Institute for Integrated Research, Institute of Science Tokyo (Formerly, Tokyo Medical and Dental University), 1-5-45 Yushima, Bunkyo-Ku, Tokyo, 113-8510 Japan; 2 Department of Nutritional Sciences, Faculty of Nutritional Sciences, Nakamura Gakuen University, 5-7-1, Befu, Jonan-Ku, Fukuoka, 814-0198 Japan

**Keywords:** Hematopoiesis, HSPC, Sox17, IAHCs, Procr

## Abstract

**Background:**

Definitive hematopoietic stem cells (HSCs) emerge within intra-aortic hematopoietic cell clusters (IAHCs) located in the dorsal aorta of the aorta-gonad-mesonephros (AGM) region during midgestation in the mouse embryo. Thereafter, HSCs migrate to the fetal liver (FL) and finally settle in the bone marrow (BM). We previously showed that the transcription factor Sox17 is expressed in IAHCs. Overexpression of the *Sox17* gene in IAHC cells induces the formation of cell clusters in vitro that resemble IAHCs and retain hematopoietic potential. In addition, a previous report showed that *Sox17*-transduced hematopoietic stem/progenitor cells (HSPCs) in the BM maintained multipotency. However, whether the ability to form such cell clusters differs among hematopoietic sites has not been fully examined.

**Methods:**

We examined whether viral overexpression of the *Sox17* gene in HSPCs derived from the AGM region, the FL, and the BM leads to the formation of cell clusters. To identify the candidate genes involved in cluster formation, we performed RNA sequencing (RNA-seq) analysis on *Sox17-ERT*-transduced cells from the AGM region and the BM cultured with or without tamoxifen. We further analyzed the ability of one candidate gene, the *Procr* gene, to support cluster formation and in vitro hematopoietic activity.

**Results:**

A large number of multilineage colonies were observed in *Sox17-ERT*-transduced tamoxifen-treated cells in all tissues examined. However, BM cells generated significantly fewer clusters than embryonic tissues under identical experimental conditions. RNA-seq analysis revealed several genes that were highly expressed in *Sox17-ERT*-transduced tamoxifen-treated cells from the AGM region. The *Procr* gene, one of these genes, was expressed in IAHCs and was found to contribute to cluster formation and to be associated with the maintenance of in vitro hematopoietic activity in *Sox17*-transduced cells of the AGM region.

**Conclusions:**

Our results revealed that the efficiency of cluster formation varies depending on the developmental origin of hematopoietic cells, suggesting that cluster formation and colony-forming activity can be partially dissociated during the transition from fetal to adult hematopoiesis. Moreover, the *Procr* gene appears to contribute to cluster formation and to be associated with enhanced in vitro hematopoietic activity in midgestation mouse embryos.

**Supplementary Information:**

The online version contains supplementary material available at 10.1186/s41232-026-00420-w.

## Background

In mammals including mice, the sites of embryonic hematopoiesis change over the course of development [1]. Hematopoietic stem cells (HSCs), which possess the long-term repopulating ability, initially emerge in the aorta-gonad-mesonephros (AGM) region at embryonic day (E) 10.5 [2, 3]. After the production of HSCs in the placenta around E12.5 [4, 5], HSCs expand in the fetal liver (FL) and finally migrate to the bone marrow (BM). In the AGM region, HSCs are observed in the intra-aortic hematopoietic cell clusters (IAHCs), which arise from the hemogenic endothelium in the dorsal aorta [6, 7]. The hemogenic endothelium is known to give rise to both hematopoietic cells and endothelial cells [8]. Whole-mount immunohistochemical analysis revealed that c-Kit, a marker of HSCs, is expressed in IAHCs. The protein expression of CD31 and vascular-endothelial cadherin (VEC), both of which are endothelial markers, and that of CD45, a hematopoietic cell marker, varies depending on the position of the cells within IAHCs. CD31 and VEC are strongly expressed in basal cells in IAHCs, supporting their emergence from hemogenic endothelium [6], whereas CD45 is expressed in apical cells [6], indicating that hematopoietic differentiation of blood cells occurs within IAHCs. Moreover, the expression levels of CD31, VEC, and CD45 differ among the HSCs in the IAHCs, the FL, and the BM [9]. Embryonic HSCs have a high proliferative capacity to supply blood cells. In contrast, BM HSCs, which exhibit altered expression of several proteins compared to IAHCs and/or FL HSCs, enter a quiescent state upon interaction with niche cells in the BM [10].

Sox17 is a transcription factor, known as an endodermal marker [11]. Sox17-deficient mice exhibit defects in gut tube formation and die during midgestation in mouse embryos [12–14]. Mice heterozygous for Sox17 show a biliary atresia-like phenotype, hepatitis with aberrant cell wall formation in the gallbladders [15–18], and female subfertility associated with implantation failure [19]. Furthermore, conditional knockout analyses have revealed that the deletion of Sox17 in the immediate postpartum period significantly reduces the absolute number of HSCs, although Sox17 does not affect the HSC numbers in the BM [20]. Whole-mount immunohistochemistry of the AGM region has shown that Sox17 is expressed in endothelial cells of the dorsal aorta and IAHC cells from the E10.5 AGM region, which are positioned close to endothelial cells in E10.5 embryos [21–23]. In our previous study [21], Sox17-transduced AGM cells maintained long-term multilineage hematopoietic reconstitution capacity, as demonstrated by transplantation assays following multiple passages in stromal co-culture. Moreover, the transcription factor Sox17 directly induces the Notch1 expression, followed by increased expression of the Notch1-downstream molecule Hes1 to sustain the hematopoietic activity [23]. Sox17 also directly enhances the expression of VEC and endothelial cell-selective adhesion molecules (ESAM), promoting the formation of hematopoietic cell clusters with hematopoietic potential [24]. Although Sox17 is not expressed in BM HSCs, enforced expression of Sox17 in adult BM-derived hematopoietic cells conferred long-term multilineage repopulating activity upon transplantation [25]. These results suggest that the Sox17-regulated molecular mechanisms active in fetal HSCs may retain latency but function in the BM HSCs [26].

We previously showed that the introduction of Sox17 into IAHC cells from the AGM region induces the formation of cell clusters with hematopoietic potential [21, 23, 24, 27, 28]. During the fetal-to-adult transition, the Sox17 expression in HSCs decreases. However, it remains unclear whether Sox17-induced cluster formation in hematopoietic stem/progenitor cells (HSPCs) is functionally linked to the fetal-to-adult transition. In the present study, we examined the formation of cell clusters and the maintenance of hematopoietic activity following Sox17 overexpression into HSPCs derived from the AGM region, the FL, and the BM. Whereas the in vitro hematopoietic activity is maintained in *Sox17*-transduced cells, their cell cluster-forming ability decreases during the fetal-to-adult transition. We compared the gene expression profiles of *Sox17-ERT*-transduced tamoxifen-treated BM cells with those of *Sox17-ERT*-transduced tamoxifen-treated IAHC cells. We also compared IAHC cells in which Sox17 was located in the nucleus with those in which Sox17 remained in the cytoplasm. From these comparisons, we identified candidate genes that were more highly expressed in *Sox17-ERT*-transduced cells where Sox17 was localized in the nucleus. One such candidate gene, the *Procr* gene (encoding endothelial protein C receptor, EPCR), was found to be expressed in IAHC cells of the dorsal aorta at E10.5 embryos. Knockdown of the *Procr* gene in *Sox17*-transduced AGM cells reduced both the cluster-forming ability and in vitro hematopoietic activity, suggesting a functional association between these properties.

## Methods

### Isolation of HSPCs prepared from the AGM region, the FL, and the BM

CD45^low^c-Kit^high^ cells, which are a component of IAHCs, were isolated from the AGM region of E10.5 mouse embryos as described previously [21]. FLs were excised from E14.5 ICR mouse embryos and suspended in Dulbecco’s modified Eagle’s medium (DMEM) supplemented with 2% (v/v) fetal calf serum (FCS). FL cells were incubated on ice for 15 min with biotin-conjugated anti-mouse CD8a, CD4, Ly6G/Ly6C, CD45R/B220, CD11b, and Ter119 antibodies (lineage antibodies, BioLegend, San Diego, CA). After washing, the cells were stained with PE-conjugated anti-mouse Sca-1 antibody (BioLegend), APC-conjugated anti-mouse c-Kit antibody (eBioscience, San Diego, CA), and PE-Cy7-conjugated streptavidin. BM cells from C57BL/6N mice were also suspended in DMEM containing 2% (v/v) FCS. After BM cells were treated with biotinylated lineage antibodies, they were stained with the APC-Cy7-conjugated anti-mouse Sca-1 antibody, the APC-conjugated anti-mouse c-Kit antibody, and the R-PE-conjugated avidin. To separate lineage-positive cells, BM cells were treated with anti-PE Microbeads (Miltenyi Biotec Inc., Auburn, CA) and passed through a magnetic-activated cell sorting column (Miltenyi Biotech). Both FL cells and lineage-negative BM cells were stained with 1 mg/ml propidium iodide (Calbiochem, San Diego, CA), and lineage^−^c-Kit^+^Sca-1^+^ (KLS) cells were recovered by fluorescence-activated cell sorting (BD Biosciences, San Diego, CA). All animal experiments were conducted in accordance with institutional guidelines and approved by the Animal Care Committee of Tokyo Medical and Dental University (approval numbers: A2018-265C2, A2019-108C4, and A2021-177).

### Enforced expression of genes in HSPCs prepared from the AGM region, the FL, and the BM

Retroviruses encoding *IRES-GFP*, *Sox17-IRES-GFP*, or *Sox17-ERT-IRES-GFP* genes were used to infect CD45^low^c-Kit^high^ cells from the E10.5 AGM region and KLS cells from the E14.5 FL for 3 h. The infected cells were then cultured with OP9 stromal cells in α-minimal essential medium (α-MEM) supplemented with 10% (v/v) FCS, 25 ng/ml stem cell factor (PeproTech, Rocky Hill, NJ), 10 ng/ml interleukin-3 (PeproTech), and 10 ng/ml thrombopoietin (PeproTech). *Sox17-ERT-IRES-GFP*-transduced cells were cultured with or without 1 μM tamoxifen citrate. Additionally, retroviruses encoding these genes were used to infect KLS cells from the BM of 10-week-old mice on the RetroNectin (Takara Bio., Kyoto, Japan)-coated dishes for 4 days. After washing, the cells were cultured with OP9 cells in the same media.

### Analyses of the cluster-forming ability and the colony-forming ability in *Sox17*-transduced cells

The exogenous gene-transduced CD45^low^c-Kit^high^ AGM cells and FL cells described above were sorted based on the GFP expression on days 4 and 7 of culture, while the gene-transduced BM cells were sorted on day 7. The sorted cells were then cultured on OP9 cells. After 11 days of culture, the number of clusters formed by the gene-transduced cells was counted. Cluster formation was evaluated by phase-contrast microscopy. A cluster was operationally defined as a visually distinguishable, compact cellular aggregate that was clearly demarcated from OP9 cells. Under conditions in which cells were spread flatly, circular or concentric colony-like structures were counted as one cluster when they formed a discrete and morphologically distinguishable unit. When partially overlapping colonies could be visually resolved as independent structures, they were counted separately. The cells were dissociated and sorted again based on the GFP expression (exogenous gene expression) for further analyses, such as RNA extraction and reverse transcription-polymerase chain reaction (RT-PCR), Giemsa staining, and the colony-forming assays.

RNA was extracted by ISOGEN (WAKO, Osaka, Japan), and cDNA was synthesized by ReverTraAce (TOYOBO, Osaka, Japan). The same amounts of cDNA were subjected to PCR using TAKARA Taq (TAKARA). Primer sequences were as follows: 5′-ACCACCCGATACCCACCTAT-3′ and 5′-GCCATGGCAGTCACCATGCT-3′ (GATA-2), 5′-GAGAGGTGGCACAACCATTT-3′ and 5′-GGGAACGTGACTGGAGATGT-3′ (c-Myb), 5′-CCAGCAAGCTGAGGAGCGGCG-3′ and 5′-CCGACAAACCTGAGGTCGTTG-3′ (Runx-1), 5′-AGGTGCAGCCACAGAACTTA-3′ and 5′-TCGGACCAATCAGAGATGTT-3′ (Notch-1), 5′-GTCATGGCCATGGTCGAGTA-3′ and 5′-CTCCTCGGCATCTTGCTGAA-3′ (CD31), 5′-GACTGGAACCAGCACGCTAACC-3′ and 5′-CGCCGTCATTGTCTGCCTCTTC-3′ (VEC), 5′-TTTATGGTGTGGGCCAAAG-3′ and 5′-CCGCTTCATGCGCTTCACCT-3′ (Sox17), and 5′-CAGCCTGGCTGGCTACGTACA-3′ and 5′-CCAGGGTGTGATGGTGGGAA-3′ (β-actin).

Band intensities were quantified using ImageJ software (NIH) with a macro file to automatically measure band intensity following background subtraction [29]. Background subtraction was performed prior to measurement. For *IRES-GFP*-transduced cells (Mock), *Sox17-IRES-GFP*-transduced cells (Sox17), and *Sox17-ERT-IRES-GFP*-transduced cells with (Tam (+)) or without tamoxifen (Tam (−)), normalization was performed in the same manner by calculating the target/β-actin ratio for each cell condition. Relative expression levels were calculated by setting the Mock condition to 1.0. When no detectable signal was observed for a target gene, the result was recorded as ND.

*Sox17*-transduced cells were transferred onto glass slides using a cytospin (Shandon, Sewickley, PA), and their morphology was examined by May-Grünwald-Giemsa staining. Additionally, *Sox17*-transduced cells were embedded in Methocult (M3434; StemCell Technologies, Vancouver, Canada) and individual colonies were counted based on morphology after 7 days of culture.

### RNA sequencing analysis

For RNA sequencing analysis, CD45^low^c-Kit^high^ AGM cells and BM KLS cells transduced with the *Sox17-ERT-IRES-GFP* gene were cultured with or without tamoxifen. After 11 days, GFP^+^ cells were sorted, and total RNA was extracted from each sample using RNeasy Plus Mini Kit (Qiagen, Germany). The RNA samples were then sent to AZENTA Japan Corp. (Tokyo, Japan) for RNA sequencing using the Illumina HiSeq/Nova seq platform and 2 × 150 bp configuration. All raw RNA sequencing data have been deposited in the DDBJ Sequence Read Archive (DRA), part of the International Nucleotide Sequence Database Collaboration (INSDC), under BioProject accession number PRJDB20795 (Run accession numbers DRR683968–DRR683971). Differential expression analysis was performed using DESeq2. Genes with an adjusted *p *value (FDR) < 0.05 were considered significantly differentially expressed. The full differentially expressed genes (DEGs) list comparing AGM Sox17^+^ and BM Sox17^+^ samples has been included as Supplementary Table S1, and the complete Sox17-induced gene set (AGM Sox17^−^ vs Sox17^+^) is provided as Supplementary Table S3. Pathway enrichment results are now provided as Supplementary Tables S2 and S4, showing Gene Ontology (GO) enrichment analyses for AGM Sox17^+^ vs BM Sox17^+^ samples and for the Sox17-induced gene set, respectively.

### Distribution analyses of Sox17 and c-Kit proteins in *Sox17*-transduced cells

After 11 days of the transduction with the *Sox17-ERT-IRES-GFP* gene into CD45^low^c-Kit^high^ AGM cells cultured with or without tamoxifen, the non-adherent *Sox17-ERT-IRES-GFP*-transduced cells were collected from the respective cultures under a microscope and transferred onto glass slides using a cytospin (Shandon, Sewickley, PA). Both the cells on the glass slides and the remaining adherent cells in the culture dishes were fixed in 2% paraformaldehyde for 10 min. They were then treated with PBS containing 1% (w/v) skim milk powder, 0.4% (v/v) Triton X-100, and 2% (w/v) bovine serum albumin (PBS-MT/BSA) for 1 h. The cells were incubated overnight at 4 °C with a rat anti-mouse CD117 (c-Kit) antibody (2B8; eBioscience). After washing three times with PBS-MT, they were stained with Alexa Fluor® 488-conjugated donkey anti-rat IgG (Life Technologies, Carlsbad, CA) in PBS-MT. Imaging was performed using the BIOREVO microscopy (BZ-X810; KEYENCE, Osaka, Japan).

### Whole-mount immunohistochemistry

Whole-mount immunohistochemistry was performed according to a previously reported protocol [23, 24, 30]. E10.5 mouse embryos were fixed in 2% PFA-PBS for 20 min and then dehydrated in methanol. After removal of the left body wall between the forelimb and hindlimb, these tissues were rehydrated in PBS and pretreated with PBS-MT at 4 °C for 1 h. The tissues were stained overnight at 4 °C in PBS-MT with rat anti-mouse CD117 (c-Kit) antibody (2B8), goat anti-mouse EPCR (encoded by a *Procr* gene) antibody (R&D, Minneapolis, MN), and rabbit anti-CD31 antibody (ab28364, Abcam, Cambridge, UK), or a rabbit anti-SOX17 antibody (EPR20684, Abcam). After washing with PBS-MT three times, the tissues were further stained overnight at 4 °C in PBS-MT with Alexa Fluor® 488-conjugated donkey anti-rabbit IgG (Life Technologies), Alexa Fluor® 546-conjugated donkey anti-goat IgG (Life Technologies), and Alexa Fluor® 647-conjugated donkey anti-rat IgG (Jackson ImmunoResearch, West Grove, PA). After washing with PBS-MT, the tissues were treated with Hoechst 33258 (Nacalai Tesque, Kyoto, Japan) and then dehydrated in methanol. These dehydrated tissues were incubated for 3 min in a 1:1 mixture of methanol and BABB (a 1:2 mixture of benzyl alcohol and benzyl benzoate), followed by a 3-min treatment with 100% BABB. The stained tissues were examined by confocal microscopy (LSM710, Carl Zeiss, Oberkochen, Germany).

### Loss-of-function analysis of the *Procr* gene in *Sox17*-transduced cells

The *Sox17-IRES-mCherry* gene was introduced into CD45^low^c-Kit^high^ AGM cells. After three passages in the co-culture with OP9 stromal cells, retrovirus-mediated introduction of short hairpin RNA (shRNA) against the *Luciferase (Luc)* gene (shLuc) or the *Procr* gene (shProcr) driven by the U6 promoter, along with a *GFP* gene driven by the SV40 promoter, was performed in *Sox17-IRES-mCherry*-transduced cells. Following three additional passages of shRNA-transduced cells, the expression of the *Procr* gene in GFP^+^ cells was analyzed by RT-PCR. The sorted GFP^+^ cells were cocultured with fresh OP9 cells and after 4 days of the culture, the number of non-adherent cell clusters was counted. Moreover, the colony-forming ability of GFP^+^ cells was assessed by Methocult (M3434). shRNAs targeted the following sequences: 5′-ACTTACGCTGAGTACTTCG-3′ (shLuc) and 5′-GTGTGGAGTTCCTGGAGAA-3′ (shProcr).

### Luciferase assay

NIH3T3 cells were transfected with a pEF-BOSE vector encoding Sox17 or the Sox17G103R mutant that lacks the DNA binding capacity, pGL3 vector (Promega, Madison, WI) containing the putative *Procr* enhancer or a point-mutated *Procr* enhancer, and RL-CMV encoding the sea pansy luciferase genes using TransIT-LT1 (Mirus, Madison, WI). After 2 days, cells were solubilized, and luciferase activities in the cell lysates were monitored using a luciferase assay system (Promega). The MicroLumat LB96P luminometer (Berthold Technologies, Bad Wildbad, Germany) was used for quantification.

### Chromatin Immunoprecipitation (ChIP) assay

The ChIP assay was performed according to a previously reported protocol [31]. The *Sox17-IRES-GFP* gene was retrovirally transduced into CD45^low^c-Kit^high^ cells from E10.5 mouse embryos. The *Sox17*-transduced cells were cocultured with OP9 cells in the medium described above. After 4 days of culture, *Sox17*-transduced cells were transferred and cultured with fresh OP9 cells. After several passages, *Sox17*-transduced cells (1.2 × 10^8^ cells) were harvested and fixed with 1% formaldehyde solution in α-MEM for 20 min. After washing with PBS, the cells were treated with lysis buffer supplemented with protease inhibitor cocktail and PMSF (ChIP-IT Express Enzymatic Kit, Active Motif, Carlsbad, CA) and cell lysates were treated by an enzymatic shearing cocktail for 5 min at 38 °C. The enzymatically digested chromatin was subjected to RNase and proteinase K treatment, followed by DNA precipitation. The DNA sample was incubated overnight with radioimmunoassay-grade BSA (WAKO)-coated Protein G magnetic beads and either normal goat IgG (control) or a goat anti-human Sox17 antibody (R&D systems). After washing, the immunoprecipitated chromatin was eluted and subjected to reverse-cross-linking and proteinase K treatment. DNA fragments were amplified by PCR using the following primers: Sox17-binding consensus sequence 1 sense, 5′-CCTAAATAATATCCGAGCTACACACGGC-3′, Sox17-binding consensus sequence 1 antisense, 5′-TAGCTTCCACATATGAGTGAGAACATGC-3′, Sox17-binding consensus sequence 3 sense, 5′-GATACTGGTCATGGTGGCTGATATCTTG-3′, and Sox17-binding consensus sequence 3 antisense 5′-CAAACAGGGTCTTACTATCCATATGAGG-3′.

## Results

### The cluster-forming ability of *Sox17-IRES-GFP*-transduced cells decreases during development

**Fig. 1:**
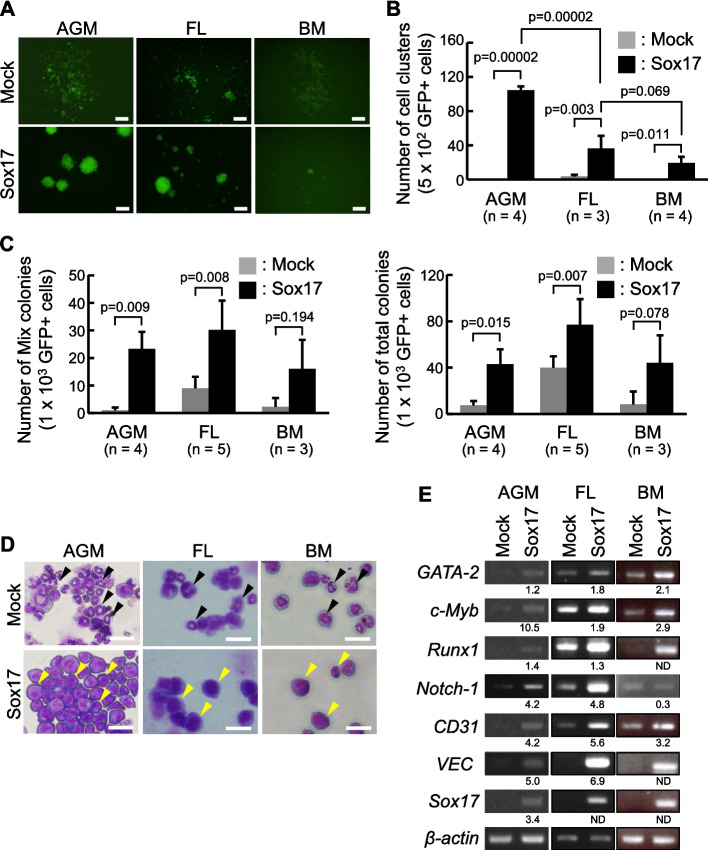
A low ability to form cell clusters of *Sox17-IRES-GFP*-transduced cells from the BM. **A**, **B** *Sox17-IRES-GFP*-transduced cells prepared from the AGM region formed cell clusters. Four days after transduction with either the *IRES-GFP* (Mock) or the *Sox17-IRES-GFP* (Sox17), the GFP^+^ cells were cocultured with OP9 stromal cells. After 7 days of the introduction, the GFP^+^ cells (5 × 10^2^ cells) were replated onto new OP9 stromal cells. After 11 days of the introduction, the number of cell clusters was counted. **A** Morphologies of *IRES-GFP*-transduced cells or *Sox17-IRES-GFP*-transduced cells derived from cells in the AGM region and cells from the FL and BM cocultured with OP9 stromal cells, after 11 days of the introduction. Bars = 100 μm. **B** Number of cell clusters in *IRES-GFP*-transduced cells or *Sox17-IRES-GFP*-transduced cells (*n* = 4, the AGM region; *n* = 3, the FL; *n* = 4, the BM). **C** Colony-forming ability of *IRES-GFP*-transduced cells or *Sox17-IRES-GFP*-transduced cells. After 11 days of the introduction, *IRES-GFP*-transduced cells or *Sox17-IRES-GFP*-transduced cells (1 × 10^3^ cells) were cultured in Methocult (M3434). The number of total and multilineage colonies was scored after 7 days of culture (*n* = 4, the AGM region; *n* = 5, the FL; *n* = 3, the BM). **D** Morphologies of *IRES-GFP*-transduced cells or *Sox17-IRES-GFP*-transduced cells were shown by Giemsa staining. Bars = 50 μm. **E** RT-PCR analysis of marker gene expression in *IRES-GFP*-transduced cells and *Sox17-IRES-GFP*-transduced cells derived from the AGM region, the FL, and the BM, cocultured with OP9 stromal cells. Band intensities from agarose gel electrophoresis were quantified using ImageJ software with a macro for automated measurement. Target gene signals were normalized to β-actin for each lane, and values are shown as relative expression compared with the Mock control below each band. Signals below the detection limit were designated as ND

We previously showed that the introduction of the transcription factor Sox17 into HSPC-containing CD45^low^c-Kit^high^ cells from the AGM region in midgestation mouse embryos maintained the formation of cell clusters with the hematopoietic ability [21, 23, 24, 27, 28]. It remained unclear whether the Sox17 overexpression could similarly sustain the cluster-forming activity in cells derived from the FL and BM. We retrovirally introduced either the *Sox17-IRES-GFP* gene or the control *IRES-GFP* gene into CD45^low^c-Kit^high^ cells of the AGM region, as well as lineage^−^c-Kit^+^Sca-1^+^ (KLS) HSPCs from the FL and BM, and these virus-infected cells were cocultured with OP9 stromal cells (representative flow cytometry profiles of AGM and BM cells in Supplementary Fig. 1A and B). On day 11 after infection, cell clusters were observed in *Sox17*-transduced cells from the FL and BM as well as from the AGM region (Fig. 1A). The number of cell clusters formed by *Sox17*-transduced cells decreased as embryonic development progressed (Fig. 1B).

### In vitro hematopoietic activity is maintained in *Sox17-IRES-GFP*-transduced cells from the FL and BM

We assessed in vitro hematopoietic activity of *Sox17*-transduced cells using the colony-forming assay. Eleven days after retroviral infection, sorted GFP^+^ cells were embedded in a methylcellulose medium. The number of total and mixed colonies containing three lineages (granulocytes, macrophages, and erythrocytes) was increased in *Sox17*-transduced cells from the AGM region, the FL, and the BM, indicating enhanced multilineage differentiation potential (Fig. 1C). By examination of May-Grünwald-Giemsa staining, *IRES-GFP*-transduced cells predominantly exhibited the morphology of granulocytes and macrophages. In contrast, *Sox17-IRES-GFP*-transduced cells showed a more blastic morphology characterized by the round nuclei and a small proportion of the cytoplasm (Fig. 1D). Moreover, the expression of hematopoietic transcription factors (GATA-2, c-Myb, and Runx1) and adhesion molecules (CD31 and VEC) was maintained in *Sox17*-transduced cells from FL and BM (Fig. 1E). Increased expression of adhesion molecules in *Sox17*-transduced BM cells was previously reported [25]. These findings suggest that Sox17 sustains in vitro hematopoietic activity, while also promoting the formation of hematopoietic cell clusters.

### The cluster-forming ability of *Sox17-ERT-IRES-GFP*-transduced cells in the presence of tamoxifen decreases with the development

**Fig. 2:**
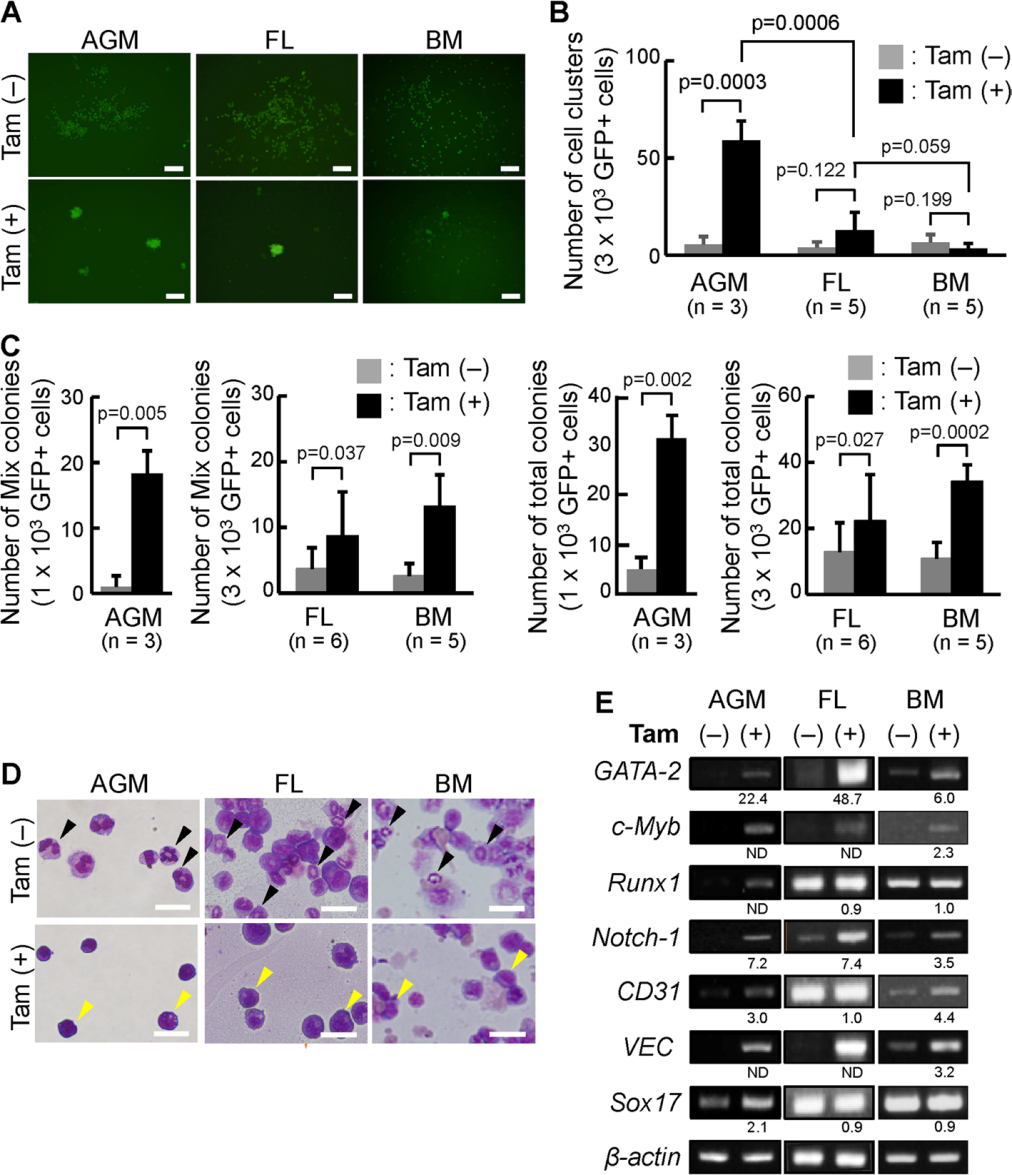
A low ability to form cell clusters of *Sox17-ERT-IRES-GFP*-transduced tamoxifen-treated cells from the BM. **A**, **B** *Sox17-ERT-IRES-GF*P-transduced cells derived from the AGM region, the FL, and the BM formed cell clusters on tamoxifen-induced nuclear translocation of Sox17. Four days after the introduction of the *Sox17-ERT-IRES-GFP* construct, the GFP^+^ cells were cocultured with OP9 stromal cells with tamoxifen. After 7 days of the introduction, the GFP^+^ cells (1 × 10^3^ cells from the AGM, 3 × 10^3^ cells from the FL and BM) were cultured with new OP9 stromal cells in media with or without tamoxifen. After 11 days of the introduction, the number of cell clusters was counted. **A** Morphologies of *Sox17-ERT-IRES-GFP*-transduced cells from the AGM region, the FL, and the BM cocultured with OP9 stromal cells with or without tamoxifen are shown, after 11 days of the introduction. Bars = 100 μm. **B** Number of cell clusters in *Sox17-ERT-IRES-GFP*-transduced cells in the culture with or without tamoxifen (*n* = 3, the AGM region; *n* = 5, the FL; *n* = 5, the BM). **C** Colony-forming ability of *Sox17-ERT-IRES-GFP*-transduced cells cultured with or without tamoxifen. After 11 days of the introduction, *Sox17-ERT-IRES-GF*P-transduced cells in the culture with or without tamoxifen were cultured in Methocult (M3434). The number of total and multilineage colonies was scored after 7 days of culture (*n* = 3, the AGM region; *n* = 6, the FL; *n* = 5, the BM). **D** Morphologies of *Sox17-ERT-IRES-GFP*-transduced cells cultured with or without tamoxifen showed by Giemsa staining. Bars = 50 μm. **E** RT-PCR analysis of *Sox17-ERT-IRES-GFP*-transduced cells derived from the AGM region, the FL, and the BM cocultured with or without tamoxifen. Band intensities from agarose gel electrophoresis were quantified using ImageJ software with a macro for automated measurement described in Fig. 1E and the Methods

Next, to examine the importance of the Sox17 transcriptional activity in maintaining the cell cluster formation and in vitro hematopoietic activity, we introduced the *Sox17-ERT-IRES-GFP* gene into CD45^low^c-Kit^high^ AGM cells and HSPCs from the FL and BM. It was previously reported that *Sox17-ERT-IRES-GFP*-transduced cells exhibit tamoxifen-induced nuclear translocation of the fusion protein [24, 32]. For the first 4 days of the culture, the cells were maintained in the presence of tamoxifen, after which they were cultured in the media with or without tamoxifen (representative flow cytometry profiles of BM cells in Supplementary Fig. 1C). After 11 days of culture, *Sox17-ERT-IRES-GFP*-transduced cells from the AGM region, the FL, and the BM formed cell clusters in the presence of tamoxifen (Fig. 2A). However, the number of cell clusters formed in response to Sox17 nuclear translocation decreased with developmental stage, with only a few cell clusters observed in BM-derived cells (Fig. 2B).

We further examined the colony-forming ability of *Sox17-ERT-IRES-GFP*-transduced cells with or without tamoxifen. An increased number of total and mixed colonies was observed in *Sox17-ERT-IRES-GFP*-transduced cells from the AGM region, the FL, and the BM in the presence of tamoxifen (Fig. 2C). The morphology of *Sox17-IRES-GFP*-transduced cells cultured with tamoxifen resembled that of cells transduced with *Sox17-IRES-GFP* gene, whereas cells cultured without tamoxifen showed morphologies similar to those of *IRES-GFP*-transduced cells (Figs. 1D and 2D). RT-PCR analysis demonstrated that the expression of GATA-2, c-Myb, and Runx1 was maintained in *Sox17-ERT-IRES-GFP*-transduced cells derived from the AGM region and the FL in the presence of tamoxifen. In BM-derived cells, the expression of GATA-2 and c-Myb was retained under the same conditions (Fig. 2E). Tamoxifen also maintained the expression of adhesion molecules CD31 and VEC in *Sox17-ERT-IRES-GFP*-transduced cells (Fig. 2E). These results indicate that the tamoxifen-inducible nuclear translocation of the Sox17-ERT fusion protein maintains the cluster formation and in vitro hematopoietic activity and gene expression in cells from the FL and BM, although this effect diminishes with development.

### The difference in the features of *Sox17-ERT*-transduced colonies, adherent colonies, and non-adherent colonies caused by the tamoxifen-inducible nuclear translocation

**Fig. 3:**
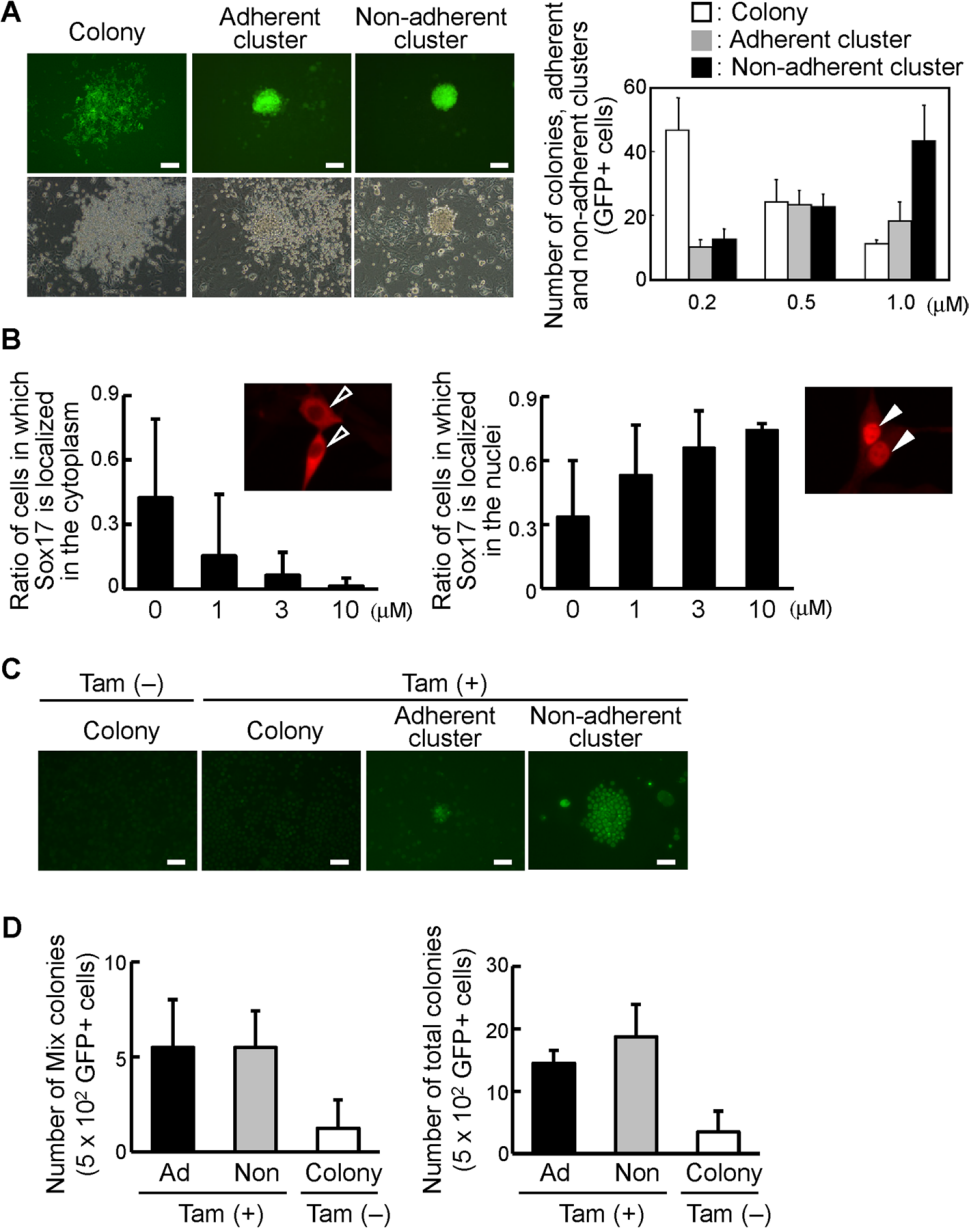
Cluster cells resulting from Sox17 nuclear translocation in the presence of tamoxifen have a higher in vitro hematopoietic activity than the colony cells in the absence of tamoxifen. **A** The frequency of *Sox17-ERT-IRES-GFP*-transduced colonies, adherent clusters, and non-adherent clusters derived from the AGM region varied depending on the concentration of tamoxifen. Morphologies of *Sox17-IRES-GFP*-transduced AGM cells, which are termed colony, adherent cluster, and non-adherent cluster were shown, after 4 days of the co-culture with OP9 stromal cells with 0.2, 0.5, and 1.0 μM tamoxifen. Bars = 100 μm. The number of *Sox17-ERT-IRES-GFP*-transduced colonies, adherent clusters, and non-adherent clusters was counted, after 4 days of the co-culture with stromal cells with 0.2, 0.5, and 1.0 μM tamoxifen. **B** Subcellular localization of Sox17-ERT protein in NIH3T3 cells with or without tamoxifen. The proportion of cells exhibiting nuclear versus cytoplasmic Sox17 localization was assessed at various tamoxifen concentrations. Arrowheads in the left photograph showed the distribution of Sox17 in the cytoplasm. White arrowheads in the right photograph indicated the presence of Sox17 in the nucleus. **C** Expression of c-Kit in *Sox17-ERT*-transduced AGM cells forming adherent and non-adherent clusters in cultures with tamoxifen. Bars = 50 μm. **D** No significant difference was observed in in vitro colony-forming capacity between adherent and non-adherent clusters. After the last 4 days of the introduction of *Sox17* genes in the culture of the AGM region with or without tamoxifen, non-adherent clusters with tamoxifen were collected under microscopy, and the remaining adherent cells containing adherent clusters were collected by pipetting. Similarly, cells from the *Sox17-ERT*-transduced colony were collected by pipetting. GFP^+^ cells were embedded in Methocult (M3434). After 7 days of the culture, the number of total and multilineage colonies was counted after 7 days of culture (*n* = 3, AGM; *n* = 5, FL; *n* = 6, BM)

Tamoxifen-dependent nuclear translocation of Sox17 in *Sox17-ERT*-transduced cells of the AGM region on the stromal cells found three types of cell morphology: colonies (the flat colony), adherent clusters (the cluster which is attached to OP9 stromal cells), and non-adherent clusters (the cluster which floats in the medium) (Fig. 3A). The number of *Sox17-ERT-IRES-GFP*-transduced non-adherent clusters derived from the AGM region was increased in a tamoxifen concentration-dependent manner, whereas the number of colonies was decreased (Fig. 3A). To examine the ratio of Sox17 nuclear translocation in a tamoxifen-dependent fashion, the *Sox17-ERT-IRES-GFP* gene was introduced in NIH3T3 cells in medium containing various concentrations of tamoxifen. The Sox17-ERT protein was detected in the cytoplasm of NIH3T3 cells cultured in the absence of tamoxifen, whereas the Sox17-ERT protein was almost exclusively found in the nucleus of the NIH3T3 cells in the culture with 10 μM tamoxifen (Fig. 3B). We showed a reduction of Sox17 in the cytoplasm at higher tamoxifen concentration, whereas the Sox17 nuclear translocation was found to increase at high concentrations of tamoxifen (Fig. 3B). These results revealed the relationship between the Sox17 nuclear translocation and the cluster formation. To examine the c-Kit expression in colonies, adherent clusters, and non-adherent clusters of *Sox17-ERT-IRES-GFP*-transduced cells of the AGM region in the presence and absence of tamoxifen, we performed the immunostaining after 11 days of culture. As shown in Fig. 3C, the c-Kit expression was not observed in the colonies, while c-Kit was expressed in adherent and non-adherent clusters by the Sox17-ERT nuclear localization in the presence of tamoxifen (Fig. 3C).

We examined the colony-forming ability of non-adherent cells and adherent cells in *Sox17-ERT*-transduced cells with tamoxifen. After 11 days of culture, the GFP^+^ cluster cells were directly recovered under the microscope, and the medium, in which some GFP^+^ clusters remained, was discarded from the dish. The new medium was added to the dish and the adherent cells containing adherent clusters and colony-forming cells were recovered after the pipetting. The colony-forming abilities of non-adherent cells and adherent cells in the presence of tamoxifen are high compared to cells in the absence of tamoxifen (Fig. 3D). There was little difference in the colony-forming ability between *Sox17*-transduced non-adherent cells and adherent cells.

### Identification of candidate genes responsible for reduced the cluster-forming ability of *Sox17*-transduced AGM cells by RNA sequencing

**Fig. 4:**
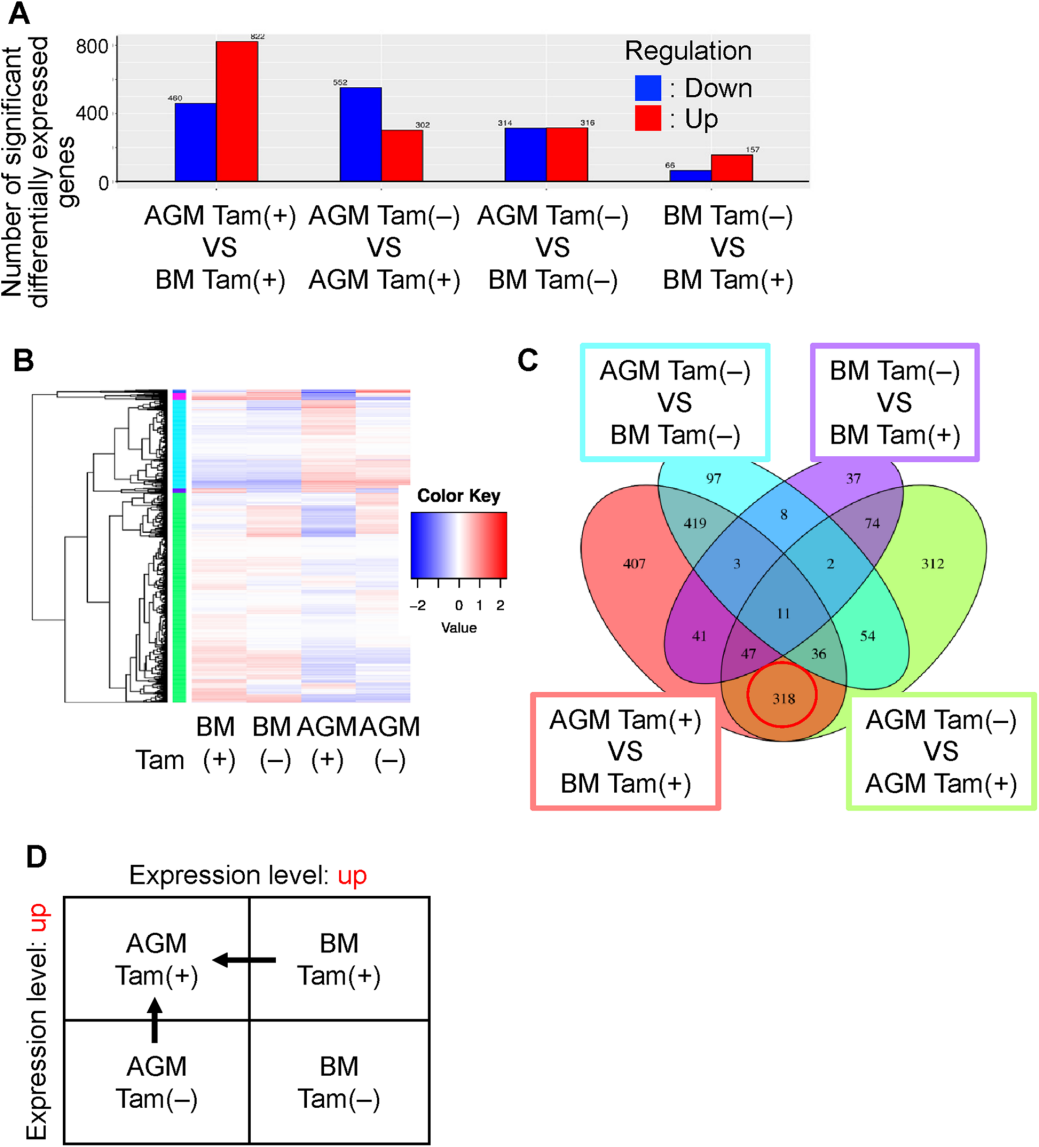
Selection of genes that were highly expressed in AGM-derived cells with the Sox17 nuclear translocation by RNA-seq. **A** The number of DEGs from *Sox17-ERT*-transduced AGM cells in the culture with tamoxifen (AGM Tam(+)) vs *Sox17-ER*T-transduced BM cells in the culture with tamoxifen (BM Tam(+)), *Sox17-ERT*-transduced AGM cells in the culture without tamoxifen (AGM Tam(−)) vs AGM Tam(+), AGM Tam(−) vs S*ox17-ERT*-transduced BM cells in the culture without tamoxifen (BM Tam(−)), and BM Tam(−) vs BM Tam(+). Red columns indicate upregulated genes, and blue bars indicate downregulated genes in the comparison between two populations. Blue columns showed genes whose expression was downregulated by comparison between two cell populations. **B** Heatmaps show the hierarchical clustering of DEGs among four cell populations. Each row represents a gene, and each column shows a sample. The color scale represents the raw Z-score of gene expression levels, ranging from blue (low expression) to red (high expression). **C** Venn diagram displaying the intersection of DEGs among the comparisons: AGM Tam(+) vs BM Tam(+), AGM Tam(−) vs AGM Tam(+), AGM Tam(−) vs BM Tam(−), and BM Tam(−) vs BM Tam(+). The numbers in each overlapping region represent the number of shared DEGs. **D** Genes that were upregulated specifically in AGM Tam(+) cells compared to both AGM Tam(−) and BM Tam(+) cells were selected for further analysis

Our present results showed that the cluster-forming ability induced by the Sox17 introduction decreased during developmental progression. However, specific genes responsible for promoting cluster formation in the AGM region and the mechanisms by which Sox17 induced the process remain unclear. To identify candidate genes involved in this activity, we conducted RNA sequencing (RNA-seq) analysis on four distinct cell populations: *Sox17-ERT*-transduced AGM cells cultured with tamoxifen (AGM Tam (+)) and without tamoxifen (AGM Tam (−)) and *Sox17-ERT*-transduced BM cells cultured with tamoxifen (BM Tam (+)) and without tamoxifen (BM Tam (−)). All RNA-seq data from this study have been deposited in the DDBJ Sequence Read Archive (DRA) under the BioProject accession number PRJDB20795, with individual Run accession numbers DRR683968–DRR683971. The number of significant DEGs identified in each pairwise comparison is shown in Fig. 4A. Heatmaps illustrating gene expression patterns across the four cell populations are presented in Fig. 4B. The Venn diagram revealed 318 overlapping genes that were differentially expressed both between AGM Tam (+) and AGM Tam (−), and between AGM Tam (+) and BM Tam (+) (Fig. 4C). From the RNA-seq data, we selected 85 candidate genes that were upregulated in *Sox17-ERT*-transduced AGM cells cultured with tamoxifen, compared to both the BM cells cultured with tamoxifen and AGM cells cultured without tamoxifen (Fig. 4D).

### Involvement of one candidate gene, the *Procr* gene, in the cluster formation with in vitro hematopoietic potential in AGM-derived HSPCs

**Fig. 5:**
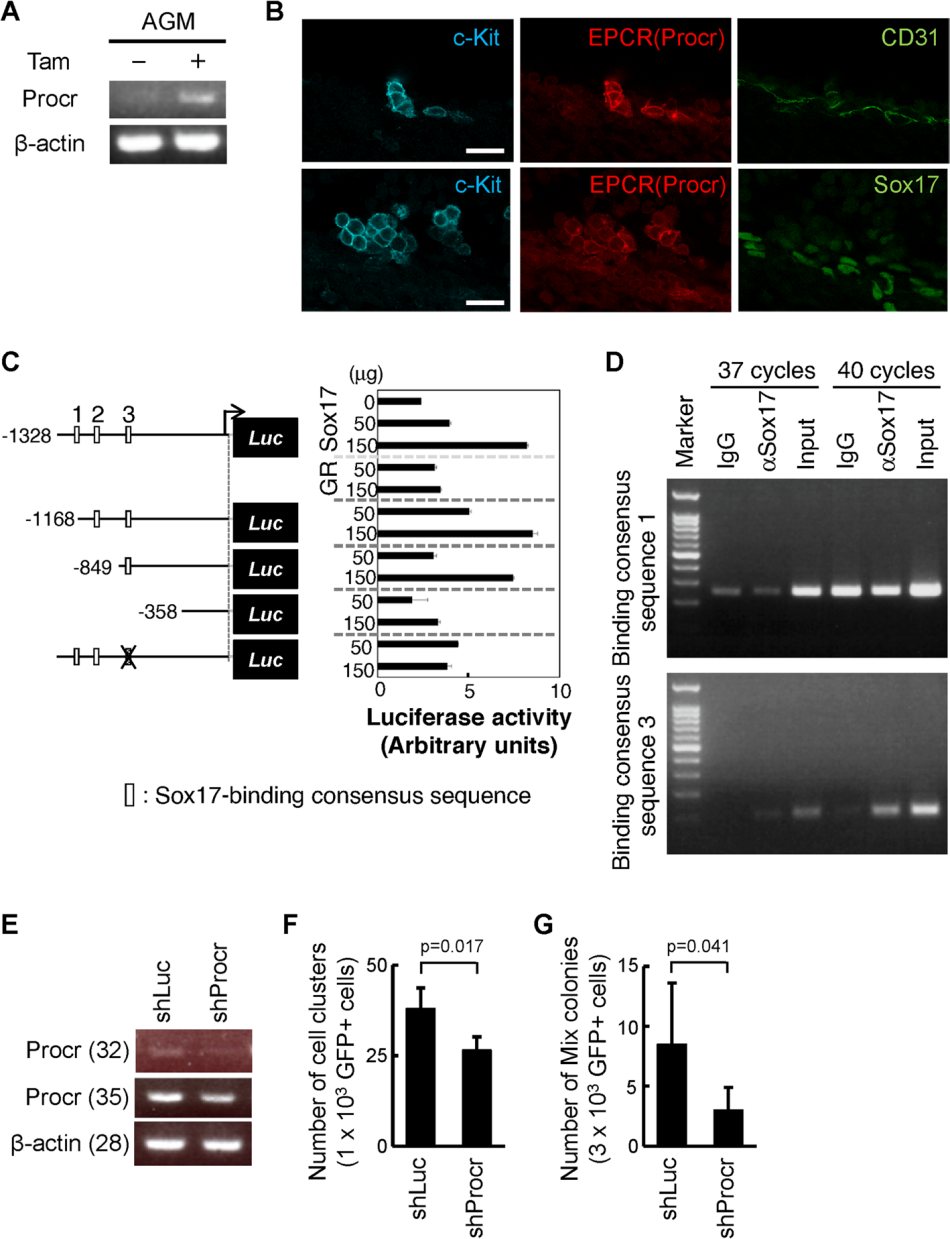
Involvement of the *Procr* gene in the cluster-forming ability and in vitro hematopoietic activity of *Sox17*-transduced AGM cells. **A** RT-PCR analysis of the *Procr* gene expression in *Sox17-ERT-IRES-GFP*-transduced cells derived from the AGM region, cultured for 4 days with or without tamoxifen. **B** Expression of EPCR, encoded by the *Procr* gene, in IAHCs of the dorsal aorta in E10.5 mouse embryos. Whole embryos were stained with antibodies against c-Kit (cyan), EPCR encoded by the *Procr* gene (red), and either CD31 or Sox17 (green). Bars = 20 μm. **C** Requirement of proximal putative Sox17-binding consensus sequence 3 for Sox17-mediated induction of the *Procr* gene. NIH3T3 cells were transfected with a pGL3 constructs containing the *Procr* enhancer fragments (1328, 1168, 849, and 358 bp) with different numbers of putative Sox17-binding sites. A 1328 bp construct containing a point mutation at site 3 was also analyzed. Reporter constructs were co-transfected with pEF-BOSE encoding Sox17 or Sox17G103R that lost the DNA binding ability. pRL-CMV was co-transfected as an internal control. **D** Direct interaction of Sox17 with the putative Sox17-binding consensus sequence 3, but not sequence 1 in the *Procr* enhancer, as determined by a ChIP assay. Digested chromatin was immunoprecipitated with either an anti-Sox17 antibody or normal goat IgG. DNA from the immunoprecipitants was subjected to PCR amplification of regions containing putative Sox17-binding consensus sequence 1 or 3. Similar results were obtained in two independent PCR analyses using samples from two independent cell preparations derived from separately obtained E10.5 embryos purchased on different days. **E** RT-PCR analysis of the *Procr* gene expression in *Sox17-IRES-mCherry*-transduced cells co-transduced with control Luc-shRNA (shLuc) or Procr-shRNA (shProcr). PCR amplification was carried out with 32 and 35 cycles for the *Procr* gene and 28 cycles for the β-*actin* gene. **F** Number of cell clusters formed by *Sox17*-transduced cells after the introduction of either shLuc or shProcr. GFP^+^ cells (1 × 10^3^), transduced with Sox17 and either shRNA, were cocultured with OP9 stromal cells. The number of clusters was counted after 11 days of the culture. **G** In vitro colony-forming ability of *Sox17*-transduced cells following the introduction of shLuc or shProcr. After 11 days of the introduction of shLuc or shProcr to *Sox17*-transduced cells, *Sox17* and shRNA-transduced GFP^+^ cells (3 × 10^3^ cells) were embedded in Methocult (M3434). The number of multilineage colonies was scored after 7 days of culture

To examine the role of these candidate genes in the cluster formation of *Sox17*-transduced cells, we focused on the *Procr* gene, which encodes the endothelial protein C receptor protein (EPCR). EPCR is known as the transmembrane glycoprotein expressed on the endothelial cells and initiates the anticoagulant pathway by activating protein C [33]. A previous study reported that EPCR, along with CD31 and c-Kit, marks HSCs in the E11.5 AGM region [34]. We first confirmed the expression of the *Procr* gene by RT-PCR and found that the *Procr* expression was upregulated in *Sox17*-transduced AGM cells cultured with tamoxifen (Fig. 5A). Next, we analyzed the EPCR expression in IAHCs in the dorsal aorta of the E10.5 mouse embryo by the whole-mount immunohistochemistry. The EPCR expression partially overlapped with that of c-Kit^+^ IAHCs and Sox17-expressing cells in the dorsal aorta (Fig. 5B). We examined the effect of Sox17 on the *Procr* gene transcription. As three putative Sox17-binding consensus sequences were observed in the upstream region of the *Procr* gene (Fig. 5C), luciferase assays were performed using enhancer constructs derived from the *Procr* gene. Analyses of deletion mutants and a construct carrying a point mutation in the putative Sox17-binding consensus sequence 3 within the *Procr* enhancer region demonstrated that the proximal consensus sequence (site 3) was essential for the Sox17-induced expression of the *Procr* gene (Fig. 5C). The direct interaction of Sox17 with the *Procr* enhancer was analyzed by a ChIP assay. The *Sox17-IRES-GFP* gene was retrovirally introduced into CD45^low^c-Kit^high^ cells from the E10.5 AGM region. After several passages, cell lysates were immunoprecipitated with the anti-Sox17 antibody, and the precipitates were subjected to PCR to assess the binding of Sox17 to the putative Sox17-binding consensus sequences 1 and 3. Sox17 directly interacted with the DNA fragment containing putative Sox17-binding consensus sequence 3, but not with that containing sequence 1 (Fig. 5D). These data support direct binding of Sox17 to the *Procr* enhancer and its transcriptional activation in vitro.

To examine the functional role of EPCR in the cluster formation, we introduced shRNA against the *Procr* gene into *Sox17*-transduced AGM cells. Specifically, *Sox17-IRES-mCherry*-transduced AGM cells were retrovirally infected with either control shRNA against control *luciferase* (shLuc) or shRNA against *Procr* (shProcr), both of which co-expressed the *GFP* gene. Eleven days after shRNA introduction, RT-PCR analysis confirmed that the *Procr* gene expression was lower in shProcr-transduced cells compared to shLuc-transduced cells (Fig. 5E). The shProcr-transduced cells exhibited fewer cell clusters than shLuc-transduced cells (Fig. 5F). Moreover, the colony-forming ability of shProcr-transduced cells was also significantly diminished relative to shLuc-transduced cells (Fig. 5G). These results indicate that reduced expression of the *Procr* gene decreases both the cell cluster-forming ability and in vitro hematopoietic activity in *Sox17*-transduced cells from the AGM region.

## Discussion

We previously showed that *Sox17*-transduced cells from the E10.5 AGM region maintained both the cluster-forming ability and the hematopoietic ability in in vitro cultures [21, 23, 24, 27, 28]. The introduction of Sox17 or tamoxifen-inducible Sox17-ERT into HSPCs from the FL and BM revealed that in vitro hematopoietic activity was maintained in both *Sox17*-transduced cells and *Sox17-ERT*-transduced cells following a tamoxifen-induced nuclear translocation (Figs. 1 and 2). We found that *Sox17*-transduced cells derived from the AGM region, the FL, and BM exhibit cluster-forming ability. This cluster-forming ability declined during development (Figs. 1 and 2).

In midgestation mouse embryos, IAHC cells expressed the c-Kit, a marker also found in HSPCs of the FL and BM [6]. Mice deleted for Runx-1, which is an essential transcription factor for definitive hematopoiesis, showed no IAHCs in the dorsal aorta and embryonic lethality [6, 35]. Conditional knockout mice of GATA2 in VEC^+^ endothelial cells also results in a reduced number of IAHCs in the dorsal aorta [7]. These results support the requirement of IAHCs for the emergence of HSPCs in the dorsal aorta. Moreover, adherent molecules such as VEC and CD31 are expressed in basal cells of IAHCs in the dorsal aorta [6, 24]. The expression of c-Kit was also observed in the cluster cells induced by nuclear translocation of Sox17, similarly to its expression in IAHC cells (Fig. 3C). In the absence of the Sox17 nuclear translocation, flat colonies were formed, while the Sox17 nuclear translocation resulted in the formation of non-adherent and adherent clusters, which were associated with increased in vitro hematopoietic activity in the AGM region (Fig. 3D). Thus, there were many similarities between IAHCs in the dorsal aorta and the cluster cells formed by the Sox17 nuclear transport.

In this study, we found that the ability of *Sox17*-transduced cells to form cell clusters decreased as development progressed. This reduction may reflect developmental differences in the cellular context in which Sox17 exerts its effects. These findings indicate that cluster formation does not necessarily equate to functional hematopoietic activity. However, differences in the composition of the input populations, particularly the higher proportion of multipotent progenitors in adult BM KLS cells, cannot be excluded and may also contribute to the observed differences in cluster formation, as these progenitors may have a reduced capacity to form cluster under our experimental condition. The BM, but not the AGM, has niche cells that support this maintenance of quiescent HSCs [36]. We hypothesized that the differences in the cell cluster-forming ability were caused by an effect of Sox17 on the induced expression of adhesion molecules [24], even though the Sox17-induced molecules are known to function in the BM [25]. To identify the molecular basis of this phenomenon, RNA-seq was performed on *Sox17-ERT*-transduced tamoxifen-treated AGM cells, which have the highest cluster-forming ability, and *Sox17-ERT*-transduced tamoxifen-treated BM cells, which have the lowest cluster-forming ability. The candidate genes were highly expressed in the *Sox17*-transduced cells containing AGM cells, which had the highest cluster-forming capacity. The *Procr* gene, which is one of their candidate genes, is expressed in IAHC cells of the dorsal aorta and is associated with the colony-forming ability in the *Sox17*-transduced cells in the AGM region (Fig. 5). The EPCR protein encoded by the *Procr* gene in HSPCs is first observed in the BM, where it marks a population enriched in LT-HSCs [37]. In the FL, the EPCR expression is also observed in HSPCs and the stem cell ability was maintained in EPCR-expressing HSPCs after a few days of in vitro culture [38]. In the AGM region of E11.5 mouse embryos, the EPCR expression level in CD31^+^c-Kit^+^ cells correlates with their long-term repopulation capacity [34]. In the present study, the *Procr* expression was elevated in *Sox17-ERT*-transduced AGM cells cultured with tamoxifen-induced nuclear translocation of Sox17 (Figs. 4 and 5A). The Sox17 expression is significantly higher in EPCR-expressing FL HSPCs compared to EPCR-nonexpressing HSPCs, and the Sox17 expression markedly decreased in both EPCR-expressing and EPCR-nonexpressing HSPCs after 1 day of in vitro culture [38]. These findings suggest a functional association between *Sox17* and *Procr* expression. Our data suggest that the *Procr* gene, as one of the Sox17-responsive genes, plays a partial role in cluster formation and is also associated with in vitro hematopoietic activity in *Sox17*-transduced AGM cells (Fig. 5F and G). It is well established that Sox17 expression decreases as hematopoietic development progresses from the AGM region to the FL and subsequently to the BM. This observation raises the possibility that the regulatory contribution of Sox17 to *Procr* gene expression is progressively reduced during development. Therefore, in the FL and particularly in the BM, alternative regulatory mechanisms may contribute to the maintenance of *Procr* expression. However, the molecular basis underlying this potential regulatory switching remains unclear.

In addition to the *Procr* gene, among the most strongly upregulated genes upon Sox17 induction were *ESAM*, *Cdh5* (encoding the VE-cadherin protein), *Tek*, and *Ptprb* (Supplementary Table S3). ESAM and VE-cadherin are key components of endothelial cell–cell junctions and play critical roles in vascular integrity and hemogenic endothelial function. Notably, previous studies have implicated both ESAM and VE-cadherin in the regulation of hemogenic endothelial properties, further supporting the biological relevance of their coordinated induction by Sox17 [24]. The coordinated induction of these junctional and signaling molecules suggests that Sox17 promotes a broader endothelial transcriptional program rather than regulating a single downstream target. Consistently, GO enrichment analysis revealed significant over-representation of extracellular matrix organization, cell adhesion, and signaling-related terms (Supplementary Table S4). These findings suggest that the *Procr* gene likely functions as part of a broader Sox17-associated molecular network contributing to endothelial identity and hemogenic competence in AGM cells.

A limitation of this study lies in its reliance on in vitro assays, including colony-forming assays and stromal co-culture systems. Therefore, cluster formation is not interpreted as a direct surrogate for long-term in vivo repopulating HSC activity. While Sox17-induced clusters are associated with increased colony-forming activity, definitive assessment of long-term self-renewal and multilineage repopulation capacity would require transplantation assays. Thus, our conclusions are limited to in vitro hematopoietic progenitor activity.

## Conclusion

The introduction of the transcription factor Sox17 into HSPCs prepared from the AGM region, the FL, and the BM maintained in vitro hematopoietic activity, but led to a progressive decline in the cluster-forming ability during development. Comparative analysis of gene expression between Sox17-ERT-transduced AGM cells and BM cells revealed that the *Procr* gene appears to contribute to cluster formation and be associated with in vitro hematopoietic activity in *Sox17*-transduced AGM cells.

## Supplementary Information

Supplementary Material 1. Representative flow cytometry patterns in viral infection.** A** In the AGM region, sorted CD45^low^c-Kit^high^ cells were infected with either the *IRES-GFP* encoding retrovirus or the *Sox17-IRES-GFP* encoding retrovirus. After 4 days of co-culture on OP9 cells, the GFP^+^ cells were recovered and used for the colony-forming assay or subsequent passages. **B** KLS cells recovered from lineage^-^ BM cells were infected with either the *IRES-GFP* encoding retrovirus or the *Sox17-IRES-GFP* encoding retrovirus. The GFP^+^ cells were similarly used for the colony-forming assay or subsequent passages. A similar gate strategy was applied to KLS cells from the FL. **C** In the analyses of the BM cells, the KLS cells sorted from lineage^-^ BM cells were infected with the *Sox17-ERT-IRES-GFP* encoding retrovirus with tamoxifen. After 4 days of co-culture on OP9 cells, GFP^+^ cells were sorted and cultured either in the presence or absence of tamoxifen. Seven days after the virus-infection, the GFP^+^ cells were collected and cultured with or without tamoxifen on new OP9 cells. After 11 days of the infection, the GFP^+^ cells were used for colony-forming assay or subsequent passages. Similar gating strategies were applied to CD45^low^c-Kit^high^ cells from the AGM region and to KLS cells from the FL.

Supplementary Material 2. Supplementary Table S1. Complete list of DEGs between AGM Sox17⁺ and BM Sox17⁺ samples. The table includes all genes tested in the RNA-seq analysis with log2 fold change (log2FC), p-value, and false discovery rate (FDR, adjusted p-value). Positive log2FC values indicate higher expression in AGM Sox17⁺ cells compared to BM Sox17⁺ cells.

Supplementary Material 3. Supplementary Table S2. GO enrichment analysis of differentially expressed genes between AGM Sox17⁺ and BM Sox17⁺ samples. Over-represented GO terms were identified based on differentially expressed genes. The table includes GO category, ontology classification (BP, CC, MF), gene counts, p-value, and FDR.

Supplementary Material 4. Supplementary Table S3. Complete list of DEGs between AGM Sox17⁻ and AGM Sox17⁺ samples (Sox17-induced gene set). The table includes all genes tested in the RNA-seq analysis with log2FC, p-value, and FDR (adjusted p-value). Positive log2FC values indicate genes upregulated upon Sox17 induction.

Supplementary Material 5. Supplementary Table S4.GO enrichment analysis of Sox17-induced genes in AGM samples. Over-represented GO terms were identified among genes differentially expressed between AGM Sox17⁻ and Sox17⁺ cells. The table includes GO category, ontology classification (BP, CC, MF), gene counts, p-value, and FDR.

Supplementary Material 6. The original RT-PCR image.

## Data Availability

Further information and requests for resources and reagents should be directed to the author: Ikuo Nobuhisa (nobuhisa@nakamura-u.ac.jp).

## Abbreviations

HSCs: Hematopoietic stem cells
IAHCs: Intra-aortic hematopoietic cell clusters
AGM: Aorta-gonad-mesonephros
FL: Fetal liver
BM: Bone marrow
HSPCs: Hematopoietic stem/progenitor cells
E: Embryonic day
VEC: Vascular-endothelial cadherin
ESAM: Endothelial cell-selective adhesion molecules
DMEM: Dulbecco’s modified Eagles medium
KLS: Lineage^−^c-Kit^+^Sca-1^+^
RT-PCR: Reverse transcription-polymerase chain reaction
Tam: Tamoxifen
EPCR: Endothelial protein C receptor protein
shRNA: Short hairpin RNA
Luc: Luciferase
ChIP: Chromatin Immunoprecipitation
GO: Gene Ontology
DEG: Differentially expressed gene
