# Supplementary material for: Ability to form Sox17-induced hematopoietic cell clusters varies among distinct hematopoietic sites during development
Source: Inflamm Regen. 2026 Apr 23;46:18. doi: 10.1186/s41232-026-00420-w (PMC13104338; doi:10.1186/s41232-026-00420-w)
Supplement: Supplementary file 1 — Supplementary Material 1. Representative flow cytometry patterns in viral infection. A In the AGM region, sorted CD45lowc-Kithigh cells were infected with either the IRES-GFP encoding retrovirus or the Sox17-IRES-GFP encoding retrovirus. After 4 days of co-culture on OP9 cells, the GFP+ cells were recovered and used for the colony-forming assay or subsequent passages. B KLS cells recovered from lineage- BM cells were infected with either the IRES-GFP encoding retrovirus or the Sox17-IRES-GFP encoding retrovirus. The GFP+ cells were similarly used for the colony-forming assay or subsequent passages. A similar gate strategy was applied to KLS cells from the FL. C In the analyses of the BM cells, the KLS cells sorted from lineage- BM cells were infected with the Sox17-ERT-IRES-GFP encoding retrovirus with tamoxifen. After 4 days of co-culture on OP9 cells, GFP+ cells were sorted and cultured either in the presence or absence of tamoxifen. Seven days after the virus-infection, the GFP+ cells were collected and cultured with or without tamoxifen on new OP9 cells. After 11 days of the infection, the GFP+ cells were used for colony-forming assay or subsequent passages. Similar gating strategies were applied to CD45lowc-Kithigh cells from the AGM region and to KLS cells from the FL. [file 41232_2026_420_MOESM1_ESM.pptx]

## Slide 1
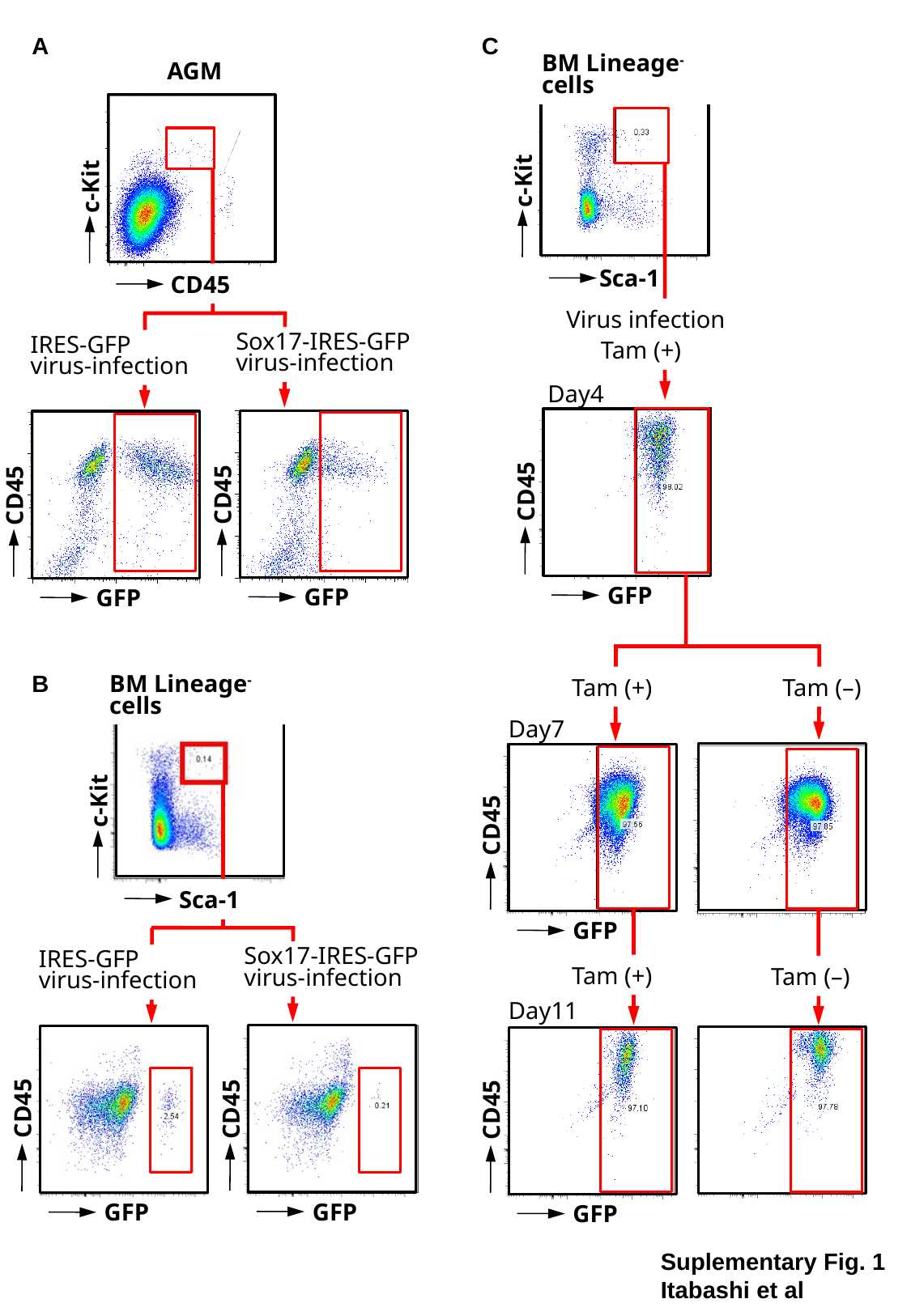

A
C
BM Lineage- cells
AGM
c-Kit
c-Kit
Sca-1
CD45
Virus infection
Sox17-IRES-GFP
virus-infection
Tam (+)
IRES-GFP
virus-infection
Day4
CD45
CD45
CD45
GFP
GFP
GFP
B
Tam (+)
Tam (–)
BM Lineage- cells
Day7
c-Kit
CD45
Sca-1
GFP
Sox17-IRES-GFP
virus-infection
IRES-GFP
virus-infection
Tam (+)
Tam (–)
Day11
CD45
CD45
CD45
GFP
GFP
GFP
Suplementary Fig. 1 Itabashi et al
