# Supplementary material for: Ability to form Sox17-induced hematopoietic cell clusters varies among distinct hematopoietic sites during development
Source: Inflamm Regen. 2026 Apr 23;46:18. doi: 10.1186/s41232-026-00420-w (PMC13104338; doi:10.1186/s41232-026-00420-w)
Supplement: Supplementary file 6 — Supplementary Material 6. The original RT-PCR image. [file 41232_2026_420_MOESM6_ESM.pptx]

## Slide 1
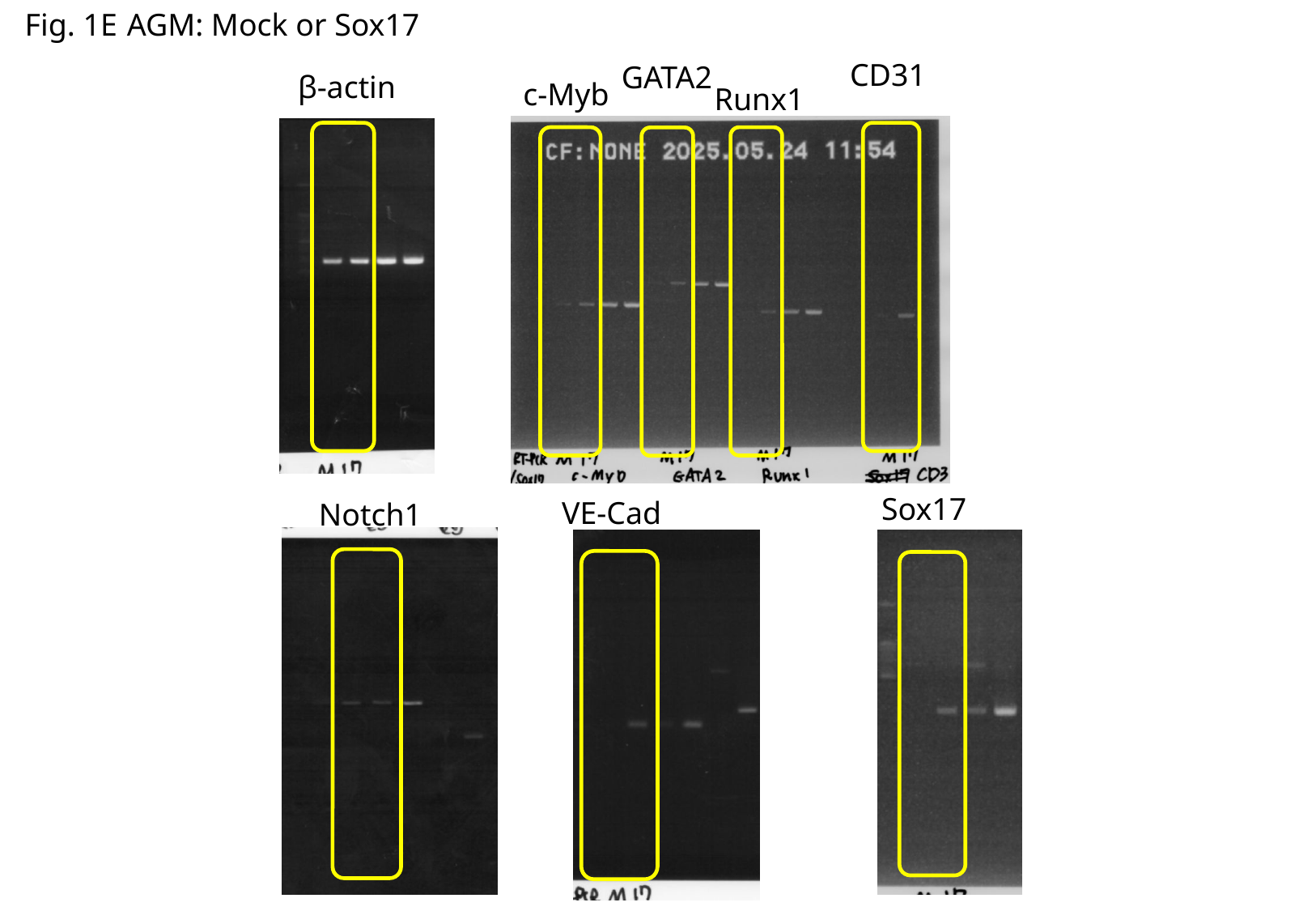

Fig. 1E
AGM: Mock or Sox17
CD31
GATA2
β-actin
c-Myb
Runx1
Sox17
VE-Cad
Notch1

## Slide 2
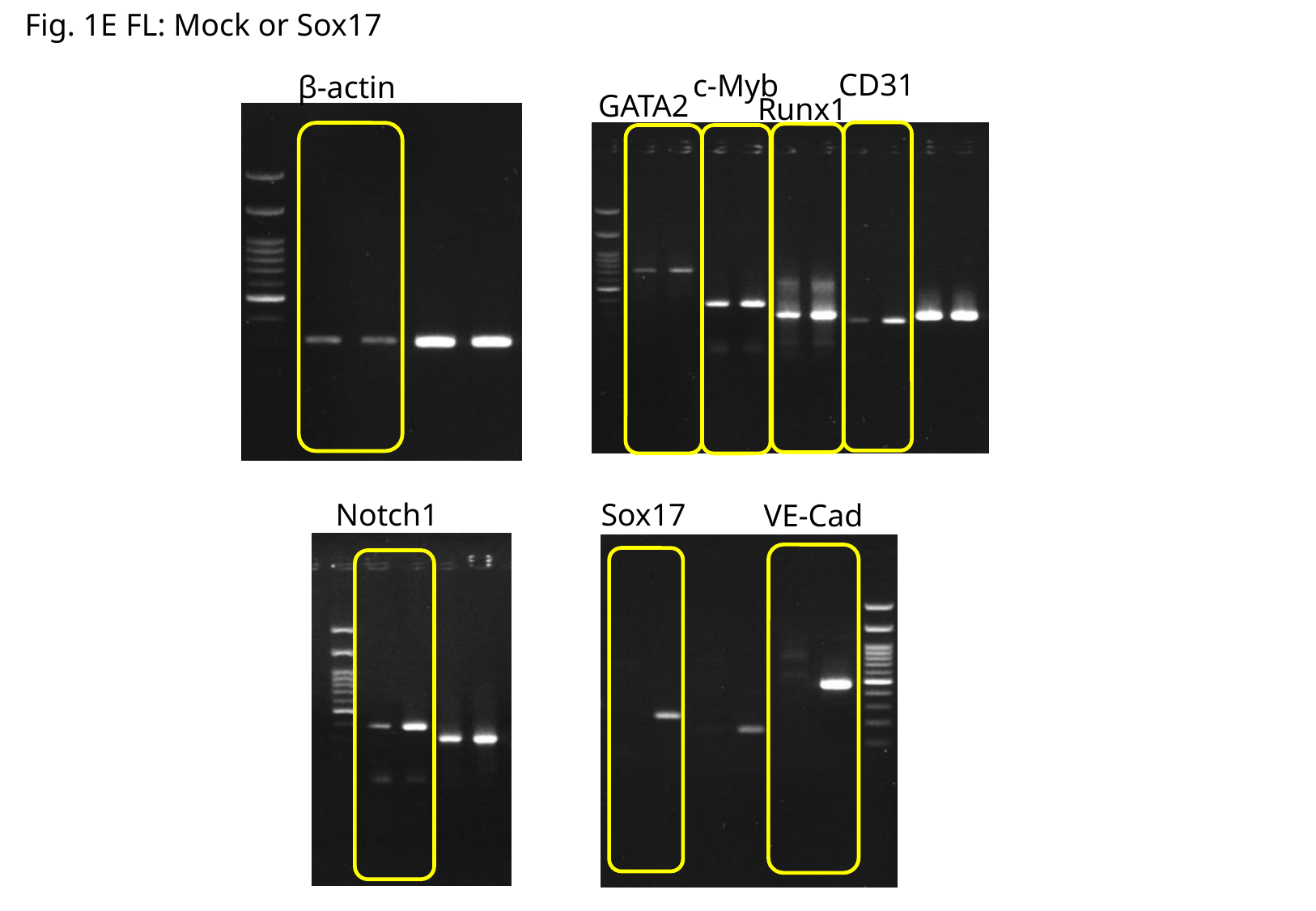

Fig. 1E
FL: Mock or Sox17
CD31
c-Myb
β-actin
GATA2
Runx1
Notch1
Sox17
VE-Cad

## Slide 3
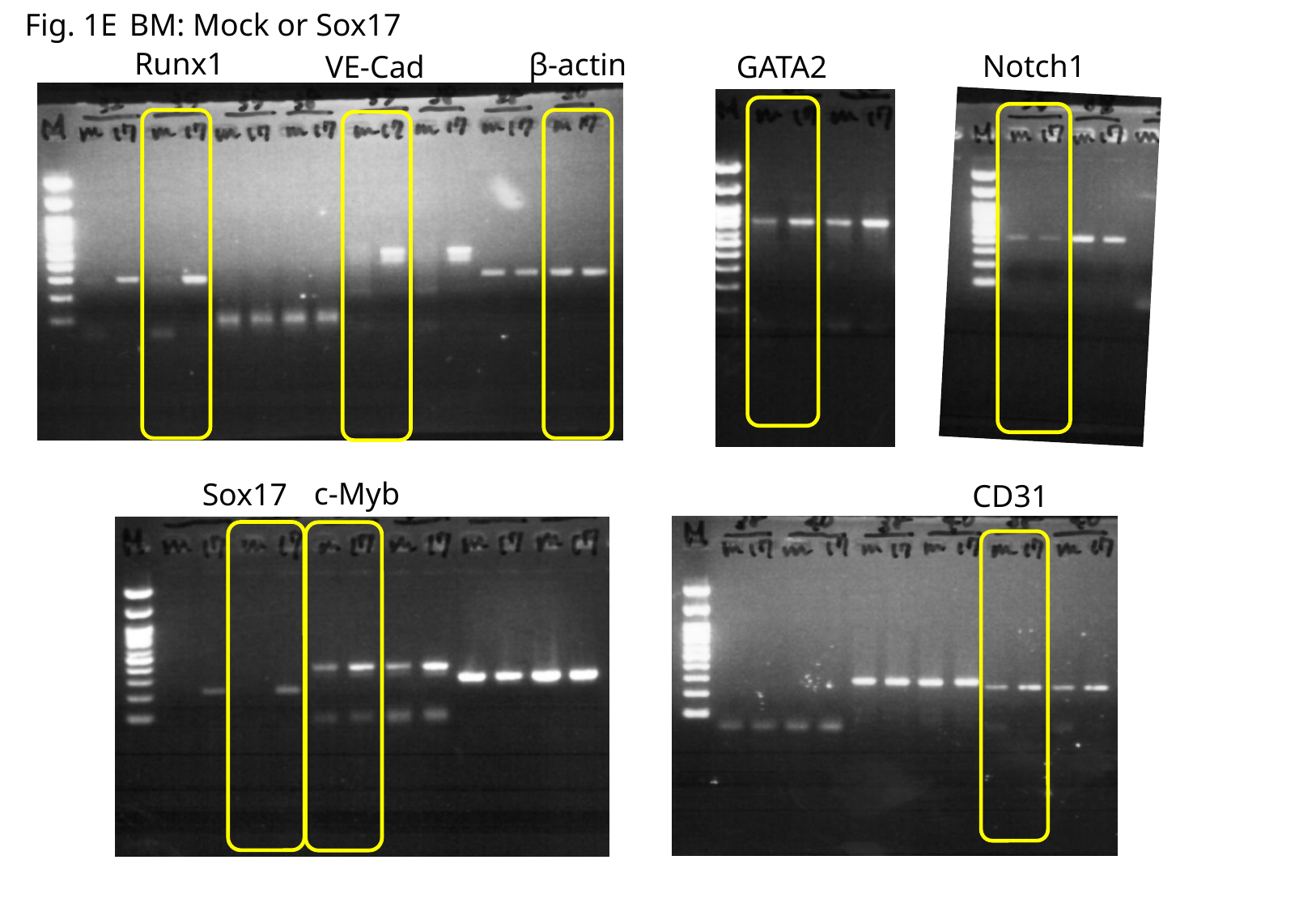

Fig. 1E
BM: Mock or Sox17
Runx1
β-actin
Notch1
VE-Cad
GATA2
c-Myb
Sox17
CD31

## Slide 4
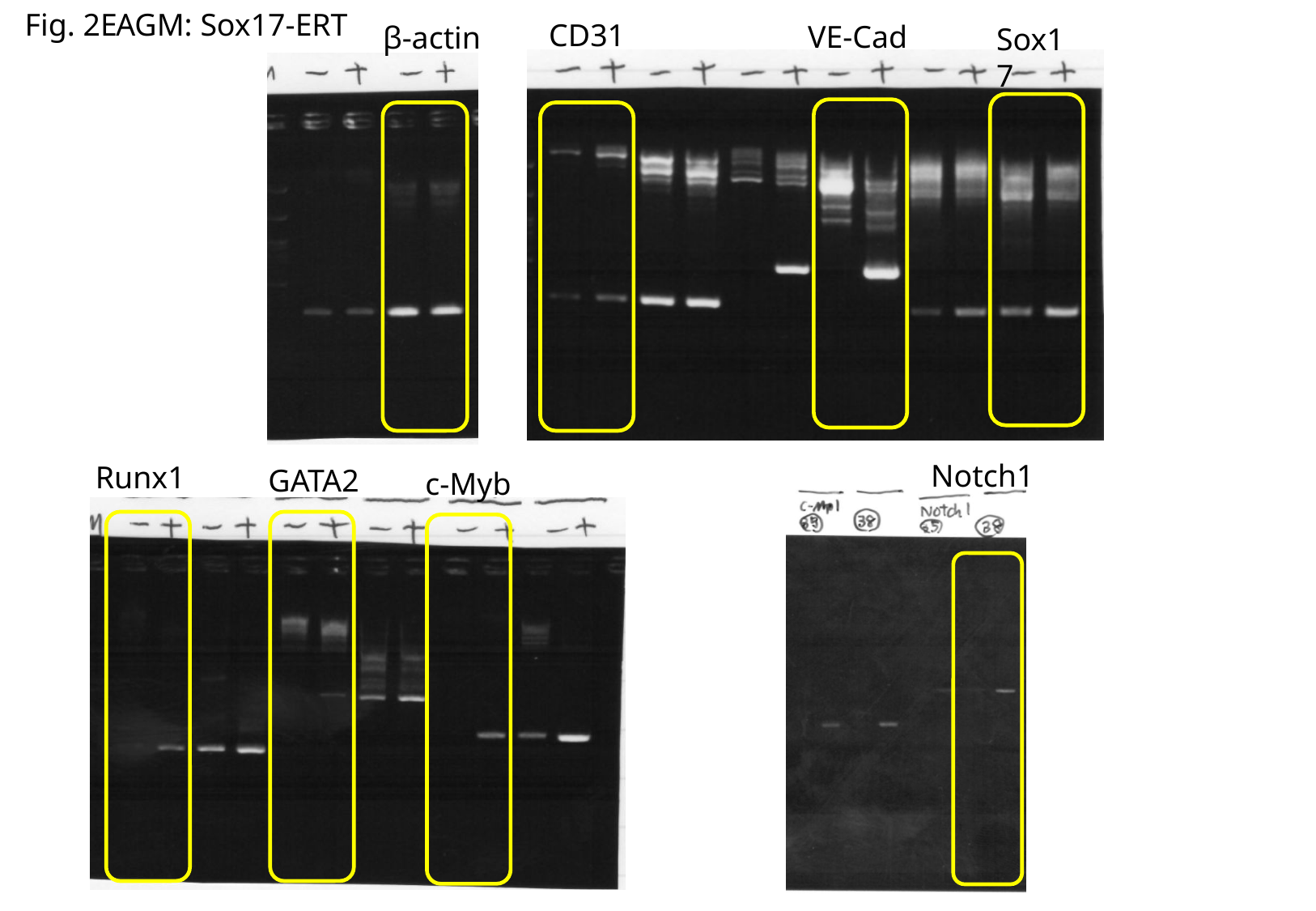

Fig. 2E
AGM: Sox17-ERT
CD31
VE-Cad
β-actin
Sox17
Notch1
Runx1
GATA2
c-Myb

## Slide 5
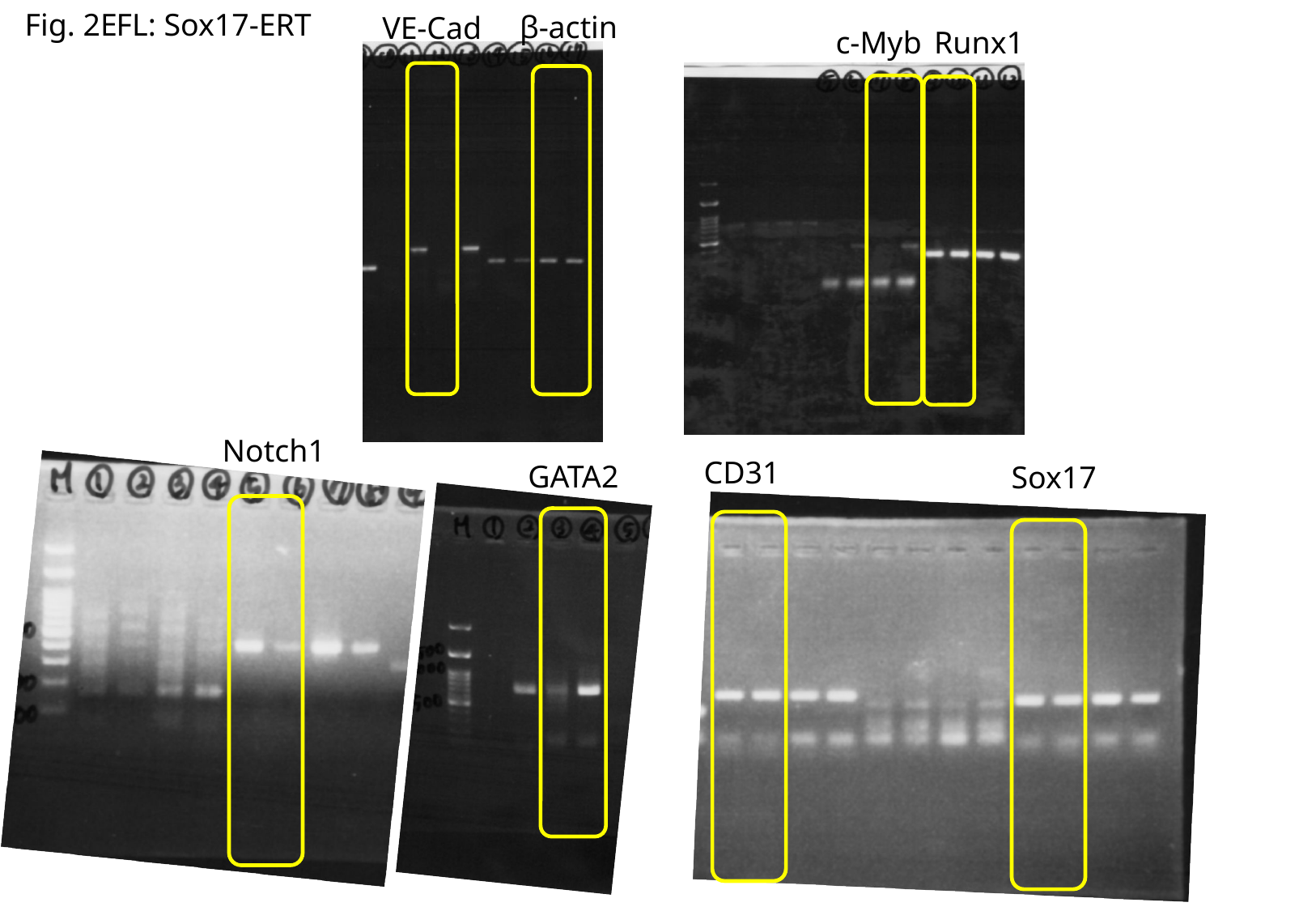

Fig. 2E
FL: Sox17-ERT
β-actin
VE-Cad
c-Myb
Runx1
Notch1
CD31
GATA2
Sox17

## Slide 6
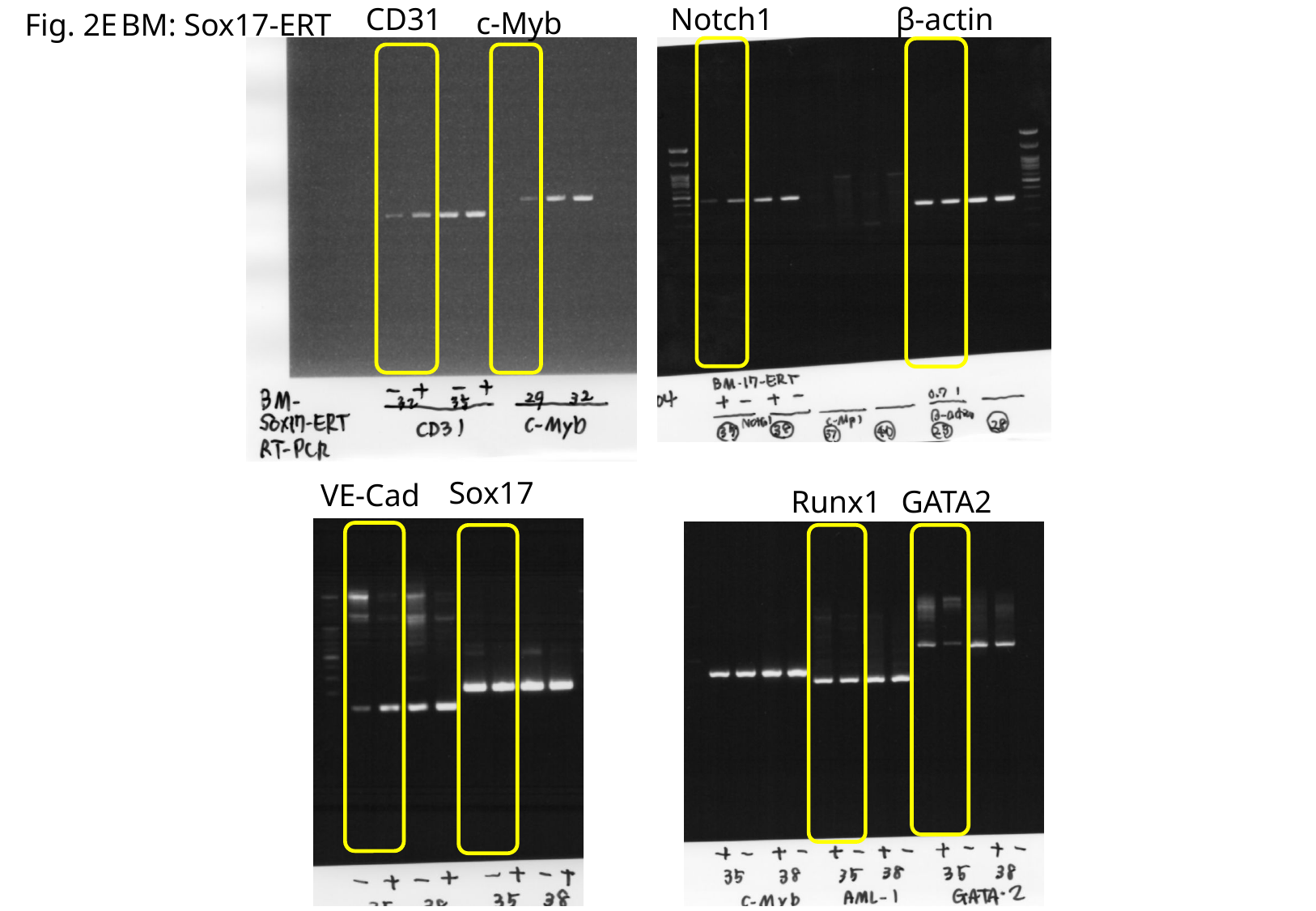

Fig. 2E
BM: Sox17-ERT
β-actin
Notch1
CD31
c-Myb
Sox17
VE-Cad
Runx1
GATA2

## Slide 7
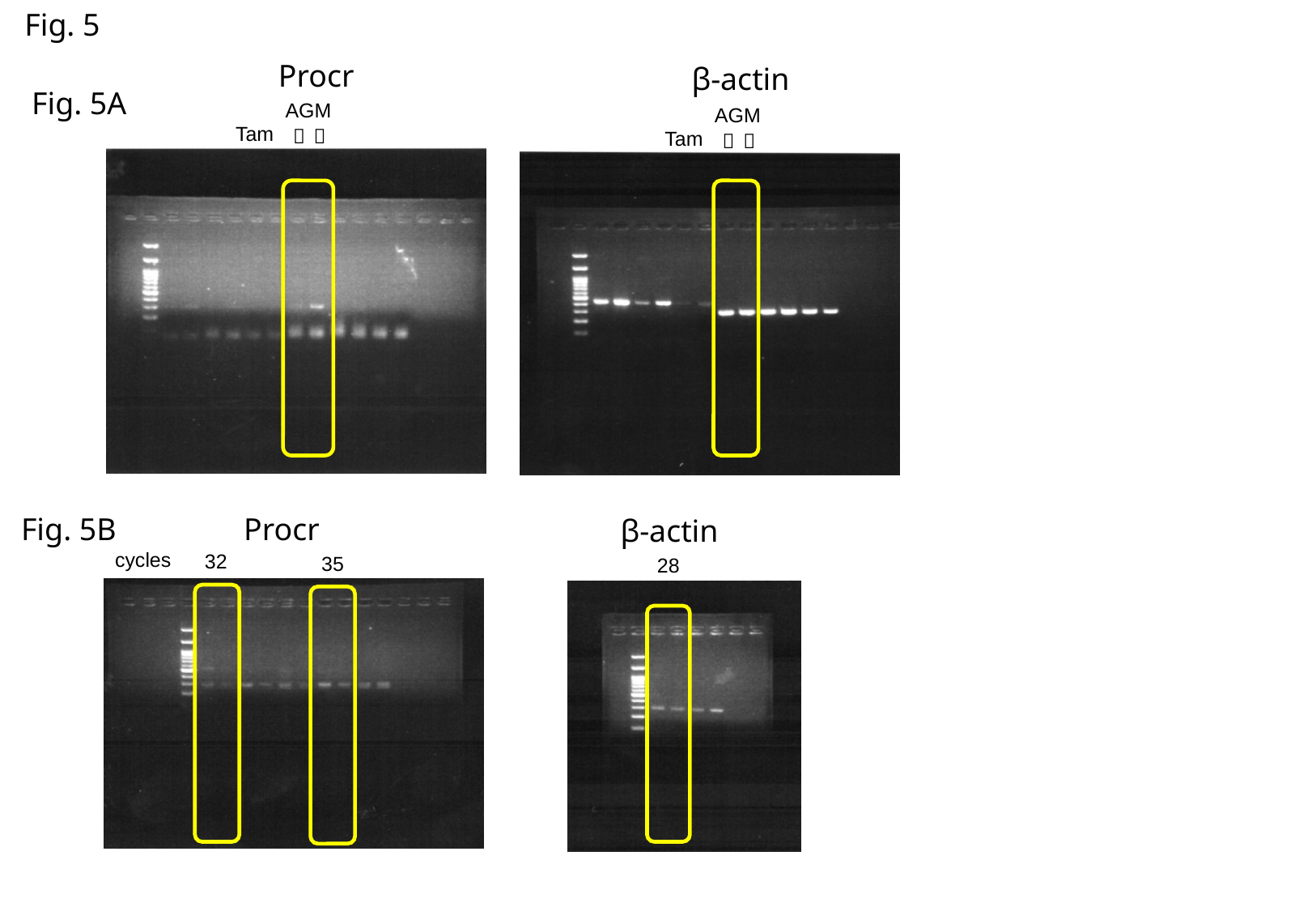

Fig. 5
Procr
β-actin
Fig. 5A
AGM
AGM
Tam
＋
－
Tam
＋
－
Fig. 5B
Procr
β-actin
cycles
32
35
28
